# Animal health emergencies: a gender-based analysis for planning and policy

**DOI:** 10.3389/fvets.2024.1350256

**Published:** 2024-04-05

**Authors:** Ellen Carlin, Claire E. Standley, Emily Hardy, Daniel Donachie, Tianna Brand, Lydia Greve, Sonia Fevre, Clare Wenham

**Affiliations:** ^1^Center for Global Health Science and Security, Georgetown University, Washington, DC, United States; ^2^Parapet Science & Policy Consulting, Washinton DC, United States; ^3^World Organisation for Animal Health, Paris, France; ^4^London School of Economics and Political Science, London, United Kingdom

**Keywords:** animal health emergencies, gender, women, disaster management planning, infectious animal disease

## Abstract

There has been increasing recognition of gender-based inequity as a barrier to successful policy implementation. This consensus, coupled with an increasing frequency of emergencies in human and animal populations, including infectious disease events, has prompted policy makers to re-evaluate gender-sensitivity in emergency management planning. Seeking to identify key publications relating to gendered impacts and considerations across diverse stakeholders in different types of animal health emergencies, we conducted a non-exhaustive, targeted scoping review. We developed a matrix for both academic and policy literature that separated animal health emergencies into two major categories: humanitarian crises and infectious disease events. We then conducted semi-structured interviews with key animal health experts. We found minimal evidence of explicit gender responsive planning in animal health emergencies, whether humanitarian or infectious disease events. This was particularly salient in Global North literature and policy planning documents. Although there are some references to gender in policy documents pertaining to endemic outbreaks of African swine fever (ASF) in Uganda, most research remains gender blind. Despite this, implicit gendered themes emerged from the literature review and interviews as being direct or indirect considerations of some research, policy, and implementation efforts: representation; gendered exposure risks; economic impact; and unpaid care. Absent from both the literature and our conversations with experts were considerations of mental health, gender-based violence, and intersectional impacts. To remedy the gaps in gender-based considerations, we argue that the intentional inclusion of a gender transformative lens in animal health emergency planning is essential. This can be done in the following ways: (1) collection of disaggregated data (race, gender, sexual orientation, etc.); (2) inclusion of gender experts; and (3) inclusion of primary gendered impacts (minimal representation of women in policy positions, gender roles, economic and nutrition impacts) and secondary gendered impacts (gender-based violence, mental health, additional unpaid care responsibilities) in future planning.

## Introduction

As the world experiences an increasing frequency of emergencies and infectious disease events affecting human and animal populations (1–3) health officials and emergency planners are working to develop preparedness and response policies, plans, and research agendas that can meet that considerable challenge.

Gender is increasingly recognized as a key determinant of infection, outcomes, and secondary effects of health emergencies, and indeed as a determinant more broadly within the disaster risk reduction field (4). Gender considers the characteristics of men, women and non-binary individuals that are socially constructed and proscribes to individuals the norms, behaviors and roles associated with their gender as well as the relationship between these groups. Gender roles are not fixed, are context specific, and can be dynamic over time. Yet a universal mainstay of gendered relations is a hierarchical power structure which produces inequalities for particular groups, including with respect to access to resources, public and private roles, decision making power and societal relations (5). Gender intersects with additional drivers of inequality and social determinants of health including sexuality, class and socio-economic status, religion, ethnicity, citizenship and disability (6).

As such, many sectors are beginning to come to terms with gender inclusions in their policy and programmatic work. Given the path dependencies that exist across emergency planning and preparedness, and the framing of particular issues of those of “health security,” “disaster management,” “veterinary” or “public health,” social issues such as the downstream gendered effects of an animal health emergency have not been considered, or have been considered of secondary importance to the immediate pathogen-related response (7). This can be attributed to the fact that those working in the response to an animal health emergency might well be trained in veterinary medicine, and thus through no fault of their own not trained to consider social impacts. This can be compounded within governments through the siloization of policy within Ministries of Agriculture or Trade for example, which may lead to policy trajectories more aligned with the consideration of economic output over that of social impacts. However, in many planning units for emergencies, this is beginning to change, with shifts to consider the downstream effects of policy interventions within crises.

To understand the extent to which policies consider gender, the United Nations (UN) uses the Gender Results Effectiveness Scale (2015) based on the Gender Integration Continuum. The idea of such an approach is to define the policy approaches according to whether it is: gender negative (a negative outcome that aggravated or reinforced gender inequalities and norms); gender blind (or gender neutral) (no attention to gender and failure to acknowledge the differential needs of men, women and marginalized groups); gender targeted (focused on the number of men, women or other genders targeted by a policy intervention, i.e., representation); gender responsive (policy addresses the differential needs of men, women and marginalized populations and focuses on the equitable distribution of benefits, resources, and status rights, but does not address the root causes of inequalities); or gender transformative (addressing cultural norms, cultural values, power structures and the root causes of gender inequalities and discrimination). Moreover, it is also important to acknowledge that gender is not the only determinant of inequalities and inequities across society. For this, intersectionality plays an important role. According to Crenshaw (6), intersectionality is “a metaphor for understanding the ways that multiple forms of inequality or disadvantage sometimes compound themselves and create obstacles”. For example, gender is one attribute which might create disadvantage, but this can be overlaid with other vulnerabilities related to race, ethnicity, age, location, socio-economic status, disability, religion, sexual identity, etc.

Research in human health emergencies has shown that gender impacts how individuals may experience outbreaks. From a primary perspective, women are more likely to be exposed to pathogens as a result of their disproportionate participation in the healthcare sector and unpaid care activities caring for sick family or community members (8). Biological and/or social factors further determine differentiation between infection and outcome rates between men and women, such as adherence to public health measures, non-pharmaceutical interventions and health seeking behaviors (9). Women also experience the downstream, secondary effects of health emergencies, which manifest through: the increased burden of unpaid care, for example, with school closures or caring for neighbors; the additional labor performed by a healthcare workforce primarily comprised of women; the increased risk of unemployment due to either the increased unpaid labor within the homes or because of the disproportionate impact on feminized sectors of the economy; reduced access to sexual and reproductive health services; or increased risk of gender based violence (7). Policies are often written gender blind, in that they do not consider the differential effects of policy and legislation on and between genders (7, 10).

Given that gender power relations and unique harms persist across all societies and economic sectors, we sought to understand the intersection of gender and animal health emergencies. We wanted to first identify gender inequities exacerbated by animal health emergencies–such as limited access to resources, imbalanced power relations in decision making, gendered health roles and community practices, cultural norms and values, as well as interpersonal experiences including gender-based violence–and second, elucidate the extent to which gender is being considered (or neglected) by animal health emergency planners.

## Materials and methods

### Literature review

We conducted a non-exhaustive, targeted scoping review seeking to identify key publications relating to gender impacts and considerations across diverse stakeholders in different types of animal health emergencies. We developed a matrix that separated animal health emergencies into two major categories: humanitarian crises and infectious disease events. Each of these were further divided into three sub-categories. Under humanitarian crises, the sub-categories were natural disasters, climate change, and disasters resulting from human activity. Under infectious disease events, the sub-categories related to the different sources of disease emergence: natural emergence; laboratory or other accidental releases; and deliberate events (including agroterrorism). The literature review targeted published information about animal health emergencies (or animal health more generally) that tied such events to these sub-categories; it did not target human health emergencies, although we do reference some human health literature in the paper as a comparator. We also identified six groupings of relevant stakeholders where gendered impacts may be experienced: animal health professionals; large-scale and commercial producers; small-scale or backyard producers; supply chain and transport workers; governmental entities and decision/policy-makers; and Indigenous communities. In addition, we considered broader socio-economic and/or community impacts as a seventh stakeholder category. Using keyword searches associated with the convergence of each type of emergency and the different stakeholder categories, we sought to identify literature relevant to each square in the matrix. We did not restrict our search based on publication type or source and included both peer-reviewed and gray/white literature in our findings. Our search was restricted to publications in English.

Identified literature was examined for both explicit and potential implicit gender considerations and impacts. We utilized a previously published set of criteria (5), developed to assess gendered impacts of COVID-19, to identify implicit gendered differences in the risks and other outcomes of animal health emergencies, based on categories such as access to resources; labor and roles; power; norms and beliefs; and laws and institutions.

### Policy analysis

To scope the policy landscape of gender considerations and impacts as it pertains to animal health and animal health emergencies, we identified key policy-making and standard-setting institutions with relevance to animal health at global, regional and national levels. We began with the three major multilateral organizations of relevance to the question of animal health emergencies: the World Organisation for Animal Health (WOAH), the Food and Agriculture Organization of the United Nations (FAO), and the World Health Organization (WHO). For national level institutions, we selected a sub-sample of national animal health or animal product institutions in countries which have faced notable animal health emergencies in recent years and are geographically diverse [foot-and-mouth disease (United Kingdom)]; African swine fever (Uganda) and avian influenza (Southeast Asia). We also considered regional bodies and their documentation, using those from the countries which have suffered recent animal health emergencies the Association of Southeast Asian Nations (ASEAN), European Union (EU) and the African Union (AU). Finally, we searched for prominent non-governmental organizations with a significant livestock or animal health mandate. We searched each institution or organization’s website and available document repositories for policy-oriented documents focused on emergency preparedness and response, as well as any policy documents that might have implications for either gender or emergencies. These documents were then reviewed for any explicit mentions of gender, particularly in the context of animal health emergencies. The team also employed “snowballing” from reviewed texts and citations, to identify additional documents. The search strategy was deliberately targeted and non-exhaustive, with the aim of highlighting key policy, governance, and programmatic documents associated with animal health emergencies and assessing the extent to which, in general, animal health emergency policies from different levels of global governance explicitly take into account gender as a consideration.

### Key informant interviews

Finally, we undertook key informant interviews to validate literature review and policy findings and inform further perspective. We identified key informants to interview about their perspectives and experiences with gender in animal health, and specifically related to animal health emergencies. Recommendations for interviewees were solicited from WOAH Gender Task Force members, identified from the program of the WOAH Global Conference on Emergency Management (11) and through snowball methods from our initial interview participants. We did not set a target number of interviews, instead aiming to reach saturation in responses. Ethical approval for research interviews was obtained by the London School of Economics and Political Science Research Ethics Committee, project approval number 188842.

Interviews were semi-structured in that responses of participants guided the interview direction (See Supplementary material S1 for the initial set of guiding questions). Interview data were recorded (with the permission of the interviewee) and transcribed through Zoom or Otter.ai. Transcripts were cross-checked for accuracy and used to match respondent quotes with key themes from our literature review and policy analysis findings.

## Results and discussion

Our review of journal literature and policy documents revealed what we consider to be a paucity of consideration of how gender relates to animal health emergencies. Compared with other domains of governance, we found only a limited literature that analyzed the gendered impacts of animal health emergencies, a trend which continued amid the policy analysis. Where gender was included in policy documents, it was typically minimal, rather than representing a gender transformative approach. Similarly, during interviews, many of the informants started the conversation with a comment akin to, “I know nothing about gender in animal health emergencies”. When probed, however, interviewees universally were able to describe detailed gendered aspects to power relations, animal husbandry, disease response, or other topics, based on the contexts in which they worked, demonstrating gender awareness. One interviewee initially noted that gender “is not something that we have actually been thinking about” (national animal production expert, African region) but on further questioning, revealed that policies and practices related to gender did exist in their settings. Interviewees with more substantial gender training confirmed that gender integration and formal awareness of disproportionate impacts is relatively nascent in the field of animal health emergencies. Nevertheless, clear themes regarding gender emerged from the literature and the interviews.

We present our key findings by thematic domains which appeared most consistently in our data: representation; gendered risks of infectious disease exposure; economic impact on women; unpaid care; and legislation and activism. We also include a section on elements that were notable in their absence in the literature and policy documents, but which policy-and decision-makers may wish to consider. Finally, we note at the end of the discussion a set of observations on the lack of intersectionality and policy differences between the Global North and Global South.

### Representation

Almost universally, our interview participants started the conversation by discussing the question of representation, either in relation to the increasing feminization of the veterinary profession at large, or the increasing number of women in their particular team or workplace. This is an important first finding, that this gender targeted reflection about numbers and proportional representation in elite workplaces or professional settings is the first notion that many working in animal health emergencies consider to be important related to gender, as opposed to, for instance, the gendered impacts of emergencies or policies.

Perhaps for non-gender experts, this is unsurprising. Gender representation is often the most visual indicator of parity, and it may be where most non-gender experts have encountered these discussions. In many countries, what was once a profession primarily occupied by men has become decidedly female. In the United States (US), one study found that 61% of United States veterinarians were female in 2018 (12).^1^ This represents a substantial gender shift among veterinarians from just 2008, when the man/woman split was even (which itself was considered a milestone at the time). The women’s share of the profession has increased by 2.2% annually while the proportion of male veterinarians is declining by 2.7% annually. Meanwhile, the proportion of United States veterinary students is also increasingly women dominated, continuing to fuel the trend in the workplace (12). Trends are similar in the United Kingdom, where, as of 2018, women comprised 60% of practitioners and 80% of students (13). One study of academia in the United States, United Kingdom, Australia, and New Zealand found that women’s representation in senior academic roles in veterinary science lags, in line with academic trends more generally (12).

In the Global South, men often still outnumber women as veterinarians, though there are clear examples of outliers. In private practice in select southeast Asian countries, women represented the following proportion of the profession: Myanmar (21%); Brunei (26%); Malaysia (45%); Singapore (70%). In public practice: Thailand (35%); Philippines (36%); Myanmar (37%); Brunei (45%); Singapore (51%); Indonesia (56%); Malaysia (74%). Many of these countries, however, have majority female student enrolment in veterinary college, predicting that the proportion of practicing female veterinarians will rise (13). There are likely additional gendered differences across the veterinary workforce between veterinarians and veterinary para-professionals, and it is likely that these differences vary by country.

The feminization of the veterinary workforce was highlighted by several interviewees, who contrasted it against past trends in their country or regions:

“I think we've only had final-year graduates for the past two years now, so it’s very young, but most are women. It’s like 70 to 80% of the students are women”.

– National animal production expert, African region

Beyond simply a question of the workforce, discussions on this feminization are also reflected in explicit policies in some countries, and indeed within institutions to engage more women in leadership in the agricultural and veterinary sectors.

“Governments [are] going out of their way to deliberately favour women [in hiring decisions]”.

– International organisation expert, African region

“There is a drive in Namibia for women to be more prominent in agriculture. So, there are quite a few women in leading positions”.

– National animal production expert, African region

“I guess working in government, they try to have an intent to have more females in senior leadership roles. Saying that in our direct cohort of directors, we have many more males. You know, we often chuckle that, you know, there's just a few females”.

– Sub-national animal health agency expert, Western Pacific Region

The United States, United Kingdom, Australia and other countries experiencing these shifts are working to assess what this dynamic may mean for the future of the profession, with potential implications for practice ownership, professional development, and animal care. The workforce dynamics of veterinary medicine are complex and related to numerous factors including gender, age/generation, and shifting demand for care. Increasing female representation in the profession may strain availability for veterinary services if the majority of the profession is vets who are disproportionately responsible for childcare or other forms of care, the trend may delay or drive down childbirth rates among women vets who view the profession as too hard to manage with childcare (14). Part-time work may complicate or require adjustments to emergency response rotas, as noted by one interviewee. As the workforce increasingly feminizes in some places, there are also changing trends related to the increasing provision of care that fathers are undertaking. Understanding these factors and how they are reshaping the profession is an open question and can elucidate how gender roles and shifts may influence response to animal health emergencies, and can be further contextualized by whether veterinary professionals versus lay responders would be expected to respond to a given event. As one interview participant highlighted:

“I've just got my fifth person going on maternity leave. It's just like, oh God… It makes it really difficult … and [in general childcare needs are] disruptive in their ability to be able to come in to respond because they can only come in and work three days where we'll do a five-or seven-day roster [during acute emergencies].. But it does mean quite a lot of disruption in the response”.

– Sub-national animal health agency expert, Western Pacific Region

Gender representation in the workforce remains location-, context-and industry-specific; one interviewee from the Western Pacific region noted that both the prawn fishing industry and poultry industry in their country were predominantly led by men, but that these patterns are changing. The interviewee noted a larger proportion of women than men working in the pig industry in that country. One interviewee noted that in contrast to the femininization of the veterinary profession in their country (Australia), the broader emergency response services, with whom animal health officials interact during epidemics and other crises, are heavily male dominated. This for some was due to a combination of the inability to deploy with childcare responsiblities working conditions of emergency deployments and the fundamental ability to do so with childcare responsibilities. Furthermore, the interviewee specifically cited working with responders from transportation services, engineering, and fire departments, and these entities being almost exclusively men. This was a trend also identified in discussions of FMD outbreaks, with the affiliated sectors involved in the response to the outbreak in the United Kingdom, with abattoirs, haulage, and incineration services being predominantly men.

Amid this discussion on representation was little consideration of the impacts of having diverse workforces and/or leadership groups for animal health emergencies. While there seemed to be acceptance that greater diversity among a workforce is overall a positive dynamic, many interviewees, particularly those speaking from a Global North perspective, seemed to be focused on ‘counting women’ rather than considering the specific value of women’s participation in decision making within these fora, the outcomes of workforce activities, and policy development on women more broadly. Conversations during the interviews of women’s participation did not lead to a consideration of whether that participation would make for more gender sensitive or transformative policy. The increasing feminization of the profession is a substantive change which requires systems to adjust in order to address the changing needs to the workforce. No participant in our interviews considered the role to be played by gender advisors in the development of gender transformative policy for animal health emergencies. This lacuna is notable, given the learnings from international development and health emergencies where it is well recognized that ‘add women and stir’ approaches do not equate to greater gender awareness in policymaking (8).

### Gendered risks of infectious disease exposure

Literature in both the human and animal health disciplines has evaluated the ways in which gender may influence risk of infectious pathogen transmission. Differential risk may arise with respect to animal rearing or care because of the kinds of animals cared for or the ways in which interactions occur. Although gender considerations are assumed to focus on the experiences of women, a truly gender aware perspective should consider how all genders, including men, are impacted by social roles. For instance, nomadic men may be at an increased risk of Rift Valley fever exposure during seasonal movements when they migrate with animals and are reliant on raw milk and uninspected meat (15). Occupational risk may also play a role in transmission of Q fever to abattoir workers, although the interplay among occupation and other factors, including gender-based exposures, is complex and intersectional (16).

Highly pathogenic avian influenza offers an interesting opportunity for gender-based analysis of risk. One study found that in Thailand, men are more likely to play a primary role in animal care at commercial poultry farms, are more likely to work in abattoirs, and are more likely to engage in cockfighting (17). Meanwhile, women handle activities like sale of dressed poultry and care of the birds on smallholder farms. Velasco et al. found that most ‘farmers and government authorities interviewed believed that there are no gender implications in the AI epidemic and crisis’ and yet many acknowledged that they think that women face more risks than men, ‘mainly because men have more access to information and training on animal health’.

This was reflected in the interviews:

“And, I think in terms of exposure for some activities that women will be involved in, which might be especially preparing food, handling of food, preparation, this puts them at high risk of things such as everything from salmonella to something a lot more potentially dangerous. So, I think that's one of the ways and in terms of vulnerability, something else which I hadn't really necessarily thought about before”.

– National animal health agency expert, European region

– [on shutting wet markets due to risk of spillover] “Because [for] some people this is their source of food and we will, even when they sell a source of money [i.e. an animal] to help their families, especially for women, that they work in the markets, or they prepare the food for their families”.

– International Organization expert [headquarters (HQ)], Eastern Mediterranean region

“And I don’t know whether there's a gender difference in risk and if that gets translated into preparedness, action. I don’t think it’s necessarily been particularly looked at”.

– National animal health agency expert, European region

The gendered interaction with animals (which is in and of itself context specific) not only impacts the risks of exposure for women but can also impact the trajectory and outcomes of animal disease outbreaks. One study of smallholder knowledge, attitudes and practices revealed that male pig farmers in Uganda had higher awareness of the importance of maintaining biosecurity during outbreaks than female farmers, likely due to greater access to training and information on disease prevention. In Southeast Asia, high levels of direct contact between women and poultry puts them at higher risk of exposure to HPAI, and their reliance on raising chickens and ducks as a source of household food and income also leaves them vulnerable to the impacts of HPAI epidemics. A 2008 WHO report noted that countries with “poor veterinary services and with high densities of poultry in backyard farms and large populations of ducks have had the most difficulty in eliminating avian influenza virus H5N1 (15). Backyard or smallholder poultry farms in Southeast Asia are often managed by women. Such family-run production efforts typically have less access to veterinary services, or educational material related to both animal and human infection prevention and control (12).

Planning for animal health emergencies, and particularly infectious disease emergencies, necessitates an appreciation of gendered exposure as well as the knowledge differentials between genders that may increase health risks.

### Women’s economic impact

A dominant gender consideration which emerged from interviews as well as policy and literature review related to the role that women play in the production and care of livestock, whether paid or unpaid, and thus the gender-responsive impact that any animal health emergency may have on women’s economic and broader familial social security.

Literature and policy discussion noted the different practices of men and women in animal/agricultural production, with men more likely to oversee larger production units and farms, particularly those with larger animals (e.g., cows, sheep), and women more likely to be in charge of animals in small holdings/backyard farms (e.g., chickens) and in general of smaller animals. For example, as an expert familiar with the context in southern Africa stated:

[in Southern Africa in a] “complete [nuclear, male-headed] family, where the father and mother, husband and wife, you will find that, in general, the man is looking more at the cattle. And the woman is looking more at the chicken”.

– International organisation expert, African region

This pattern is furthered in Miller’s investigation into cultural norms around ownership. Gendered expectations and social responsibilities may contribute to the species of animal and the intentions behind having them. For example, women may keep livestock for directly feeding children, whereas men may seek out livestock as an insurance policy or display of wealth (18). Women also deal directly with manure, feeding, cooking, bones, and internal organs of animals, which both accelerates the transmission of zoonotic diseases and limits their economic mobility as the tasks concern tactical care rather than strategic farm operations (18).

This is the first gendered differential which is important: it implies that men are more likely to be working with animals in relation to capital (i.e., receiving income in return for their animal caring activities), while women are more likely to be looking after animals as a source of food or income for their immediate family, rather than as an economic activity. This is important as it implies that animal health emergencies will differentially affect household and child calorie consumption and food security depending on the species affected, with those diseases affecting poultry more likely to impact child food intake than those of cattle. This differential between the roles of men and women within farming households extends across our research. Even in U.K. literature and policy documents on the 2001 FMD outbreak, women are often seen as farmers’ wives, rather than as farmers themselves (19).

As stated by the same expert as above:

“The women will be mainly in control of the finances from the small stock and the chickens. But the husband will always give them money”.

– International organisation expert, African region

This clearly displays the economic power differential in traditional livestock rearing households in this setting and means that women may be more vulnerable to any economic shocks experienced as a consequence of a pathogen destroying their animals or being culled preemptively. For example, an unregistered smallholding or farm may not be eligible for reimbursement for lost earnings. Moreover, even women who are not involved in animal health or livestock rearing could be impacted if quarantines place limitations on economic participation in other fora, such as other forms of retail at livestock markets. One interviewee told us that in their experience, it was almost always men coming forward to receive cost replacements during animal health emergencies, even though this dominance of men may not represent the labor being done to address emergencies. Another interviewee highlighted that women’s assets are more likely to be sold first in the event of a crisis. We found some instances of the government offering other forms of support to households during animal health emergencies, such as provision of food packages, which, noting the recognized relationship between women’s economic security and household calorie intake, might be unintentionally gender-responsive:

“And then we have the food packages, we even had them during COVID. We know that some of these people will not be able to earn a living … but I'm saying this because when [the] government is doing this at the back of his mind, it has in mind single-headed families, and most of the single women-headed families, there are very few men single families”.

– International organisation expert, African region

However, other interviewees suggested that national efforts to provide in-kind support during emergencies were routinely gender-blind, with the priority being meeting pre-defined criteria and doing whatever was necessary to contain the outbreak and prevent further spread:

“It doesn't matter who's asking for the money, if it needs to be paid for maintenance of our animal health, status, or confinement, or of our outbreak, or whatever it doesn't matter”.

– National animal production expert, African region

Importantly, differential interactions between men and women in the care and ownership of animals during routine times, and who comes forward for reimbursements at a time of an emergency, may further reveal power relations between men and women in households and on farmsteads: that men are more likely to participate in public interactions with government actors, regardless of levels of contribution to work. In contrast, one interviewee noted that in the case of prawn fisheries in Australia that have been suffering from white spot disease, the men are “too emotional” to engage with government responders, and it is often their wives who were serving as the spokesperson for the family or business. Thus, the specific nature of the health emergency and the sociodemographic context can influence gendered power structures and who in a (cis-gendered heterosexual) relationship is in a position of financial power related to the emergency.

The way that compensation is provided during crises also reveals a consideration of who is able to access resources during times of crisis, predetermined by household bargaining and socio-cultural gendered norms. Systems of compensation vary by country, industry, and setting; for example, in Australia, compensation is strictly targeted toward replacement of any lost or destroyed assets. In other settings, such as Botswana, compensation is split between 70% restocking and 30% cash. We were unable to obtain any data on how farmers were spending cash money when received, and whether this varied as to whether men or women received the funds. One interviewee noted there was consideration as to whether men used the monies received to replace herds or to retire during the FMD outbreak in the United Kingdom. There are gendered differences in household payments both within smallholder farming in general (20) and in other sectors such as conditional cash transfers (21).

### Unpaid care

Women worldwide absorb the burden of unpaid care across households and communities. This is as likely to be true in animal health and care, both directly and indirectly. As was established in our literature review, women are more likely to provide the care to smallholdings of livestock, or backyard farms which serve the purpose of feeding a family. This unpaid work is vital to the food and/or financial security of an individual household, but due to a lack of gender responsive or gender transformative policies and practices, women are unlikely to be compensated for their time for such work. As discussed above, this may have economic impacts during an animal health emergency if this labor is not only unrecognized as an important feature in the livestock production system, but women are not compensated for any associated loss. In the case of infectious disease, it is possible that such interaction could lead to greater concealment of outbreaks given the limited incentives offered to these women, thus increasing the risk of pathogen spread.

Women’s unpaid labor during animal health emergencies extends well beyond that of care of backyard animals. Women provide the lion’s share of household management as well as childcare in routine times, and this may be particularly true in traditional farm settings:

“In the agriculture sector, [it] is always the man [who] runs the business. Always got a female, which is normally the wife [of] the house that is helping with the house, is helping with work, is helping in the decision making”.

– International organisation expert (HQ), European region

This burden of unpaid household management and childcare may well increase during times of an emergency. For example, if a male farmer is working additional hours to manage the spread of a disease, or negotiate with officials as to the process, protocols and mechanisms for responding to an outbreak, then this in turn may imply additional time that women will be in the home caring for children. This may have additional unintended consequences of forfeited additional income or educational advancement (such as for women who may work or study in the evenings while their partners care for children, or indeed for girls’ education who support in animal rearing or livestock). Furthermore, if mobility restrictions are put in place across communities to mitigate against a pathogen’s spread, this may have an impact on children’s routine activities such as school attendance, and result in additional time in the home.

Moreover, women are more likely to perform the function of family and community support, particularly during times of crisis. As is increasingly recognized in emergency management, women in male-headed households provide the function of supporting and providing services which are otherwise lacking during times of crisis. In an animal health emergency, this would be no different, whether supporting partners, friends and neighbors who have lost their flocks/herds or who are facing government action to mitigate the spread of disease. As one interviewee put it in reference to the current prawn fishing crisis in Australia, women are “supporting their husbands and sons” during the emergency. In different settings, such support could be both in physical format, such as contributing to the increased labor on farms at a time of crisis, or through the feeding farm workers working long hours, or emotionally through picking up the mental load of their other labors, not to mention the emotional support they may require. Yet, beyond references made by interviewees, we were unable to find empirical evidence of such informal labor that women are providing in communities during periods of acute animal health emergencies, or policies that recognize this unpaid labor. Moreover, while we did hear that mental health concerns are beginning to be recognized for farmers, this did not extend to the emotional toll of their wives and daughters living the secondary realities of such emergencies.

### Intersectionality

While this analysis is dispersed amid the other domains of analysis, it is important to highlight the differences that we identified in how gender was considered across a range of intersectional drivers of inequality. The literature, policy publications and interviewees often recognized that women were not a homogenous group, but that there were notable differences in the relationship between gender and animal health emergencies on geography, religion, socio-economic position, cultural norms, and beyond. These differences are important when designing policies to be gender sensitive, as gender mainstreamed policies may unintentionally disadvantage particular groups of women while supporting others.

This research was designed and implemented to analyze the interaction between gender and animal health emergencies. Thus, it was not designed to be an intersectional analysis, and so we did not systematically collect data points on intersectional effects. This being so, we do not present here an exhaustive summary of intersectional challenges for emergencies, but a flavor of intersectional themes that emerged organically. Further research should consider this expressly.

The literature review demonstrated the intersection between poverty and impacts experienced by women in animal health emergencies. Female-headed households in South Africa face greater food insecurity than male headed households (22). Among 137 Indigenous interview participants in South Africa, Mtileni et al. found that intersectional factors like age, gender, production system, wealth and nutritional supplements influence the spread of disease and profit derived from chicken sales (23).

One interviewee noted a difference in women’s participation in the animal health workforce dependent on nationality and/or ethnic group within the same location—that the poultry industry is predominantly men at large, but within particular social groups, poultry is a woman’s profession.

One interviewee expressly recognized the fact that thinking about gender is too myopic a lens to consider the range of impacts that women experience in and around animal health emergencies:

“When you were talking about gender equity, we’re talking about cultural equity and social equity as well”.

– Europe-based NGO expert with experience in African region

Indeed, this nuance is an important caveat to all of this report and research. Gender is context specific and does not exist in a vacuum. Thus, considering gender means also considering a range of intersecting variables which render discrimination or barriers for particular groups at the expense of others.

One area important to consideration of gender and emergencies for which we did not find any consideration was of lesbian, gay, bisexual, transgender or queer (LGBTIQ+) groups within animal health emergencies, either in terms of representation within the industry, or of secondary effects of outbreaks and the policies introduced to mitigate against them. We also note that for the most part in this article, we have based our analysis on cis-gendered heterosexual households. As with other areas of governance, this does not mean that there is no differential impact on other genders group, but that we were unable to identify any data collected to date to meaningfully understand the experiences of these groups.

### Global north/south differences

One further key finding from this research is the difference in recognition and consideration of gender and animal health emergencies between the Global South and the Global North. The literature, policy and interviews revealed a more progressive gendered recognition of animal health emergencies in the Global South. This appeared both in general (i.e., through the policy review of international organizations, and with interviews), and also in a contrast between the case studies considered here between ASF (such as in Uganda) and FMD (United Kingdom).

This differentiation is multifaceted. Firstly, there is a temporal gap between the analysis and consideration of the FMD outbreak in the United Kingdom and more recent ASF outbreaks in Uganda. The United Kingdom FMD outbreak emerged in 2001 when gender awareness and gender mainstreaming in policy was almost non-existent. The political context of the outbreak and the lack of a contemporary gendered lens cannot be forgotten in revisionist consideration of the outbreak. Secondly, although ASF is endemic in Uganda and there is no singular, acute outbreak to refer to, the increased recognition of gendered impacts have been reported and/or published more recently. Thus, the timing of the outbreaks may be responsible for greater gender integration. Thirdly, we cannot divorce the activities in gender and animal health emergencies in low-and middle-income countries from the broader international development and gender agenda which has dominated much of the academic considerations, policy development and practice for the last 20 years. It might be that many of the policies and programs that appear to be more gender sensitive than those elsewhere in the world are a secondary effect of a broader discourse around gender across these governments. Fourth, it might demonstrate a more successful civil society or improved social relationships between the public and government in gender progressive locations. This advocacy may have shifted the discourse around animal health emergencies to include gender. While we can only speculate on the reasons for the differences in gendered considerations across north and south geographies, we believe that this discrepancy is an important observation.

### What’s (surprisingly) not in the findings

When considering what our three types of data revealed about animal health emergencies and gender, we were surprised that there was no reference to issues which are ever-present in gender analysis and consideration in other spheres of governance.

#### Gender-based violence

The critical consideration absent from the literature is gender-based or domestic violence. Gender-based violence can include sexual, physical, mental and economic harm inflicted in public or private (24). It is estimated that one-third of women have experienced physical and/or sexual violence by an intimate partner at one point in their lives (25). Rates of gender-based violence increase during times of crisis, including civil unrest, natural disasters, conflict and other complex emergencies. The relationship between health emergencies and domestic and gender-based violence is also now well established as is the inter-relationship between economic insecurity and such forms of violence (26, 27). Given that animal health emergencies are a form of crisis, and can have significant economic implications on farmers (who are usually men), there may be corresponding increases in rates of gender-based violence during both the acute phases of an animal health emergency and the recovery period.

However, there was no consideration of gender-based violence in the literature or in the policy documents reviewed.^2^ When questions relating to violence were asked to interview participants, no respondent had any knowledge of any forms of gender-based violence during their professional experience working in such emergencies, though one interviewee noted that threats and anger toward veterinary officials, which sometimes spilled over into violence, was a typical response from community members, especially men, during fraught interaction amid emergencies.

However, given the clandestine nature of gender-based violence in particular, and the notorious under-reporting, it is not a surprise that those in positions of power in a health emergency, such as government advisors and or veterinarians that we interviewed, would unlikely be made aware of such violence or that animal health emergency officials may not be looking for it. Secondly, because of universal paucity of data on gender-based violence, noting the challenges of collecting such data, it might be that such data do not exist, as they are not seen as a vital data need during stages of an animal health emergency.

Gender-based violence is likely occurring during animal health emergencies, whether it is identified or not. Not only should the animal health emergencies sector be aware of increased risks of violence, but front-line workers in emergencies could also provide a useful mechanism for identifying and supporting those subject to violence. During the COVID-19 outbreak, noting that people confined to their homes were not able to access the same forms of public interaction and social support, new processes were trialed. In the Czech Republic, postmen were trained to identify violence within households, given they visit most households daily and may be the only external interaction women have (27). Veterinary workers could be trained in similar methods to detect if a woman may be subject to violence and provide alert to security and/or domestic violence support services if identified when on home or farm visits.

#### Mental health

There are likely a myriad of mental health impacts of animal health crises. Literature has identified the mental health challenges and increase risks of suicide during major emergencies (28, 29). Yet, these do not disaggregate the risks of mental health concerns by gender, meaning it is impossible to entangle the differential experiences and risks experienced by men and women during the studied crises. However, as we know from other emergencies, different genders experience emergencies and their associated mental health challenges in diverse ways.

We learned from several interviewees that in settings such as Australia and southern Africa, social workers are deployed by the government to communities affected by animal health emergencies, recognizing the psychological impact that animal losses can impart. These counseling services are offered to all, but there may be gendered uptake:

“And somehow you find most women going for counselling. I think men don’t like [it]”

– International organisation expert, African region

The same interviewees noted similar observations among veterinary professionals, who are also provided with counseling after participating in outbreak responses, for example related to FMD. Although the services are “open for everybody,” the interviewees said:

“You will find that it is mostly the lady officers who are going for counselling. I think in our setting, women will naturally like somebody to listen to or to share”.

– International organisation expert, African region

“Yeah, probably more often females will reach out and say, ‘I’m not okay with this’”.

– Sub-national animal health agency expert, Western Pacific Region

These responses reflect underlying differences in gender norms related to care-seeking and appearance of vulnerability. The latter interviewee noted that in their setting, this difference was well-recognized, and that leaders were often ‘more concerned’ with male responders due to their lower likelihood to seek out or accept psychological help when in need, though no specific policies to encourage male uptake of social or mental health services were in place.

### Seeds of change

The United Nations Charter reaffirms “the equal rights of men and women. The Convention on the elimination of all forms of discrimination against women (CEDAW) was adopted in 1979. The Beijing declaration and platform for action in 1995 further cemented this global commitment toward gender mainstreaming across all domains. Such global policy push for gender equality is also reflected within the United Nations Security Council (UNSC) resolution 1,325 that “urges Member States to ensure increased representation of women at all decision-making levels in national, regional and international institutions and mechanisms for the prevention, management, and resolution of conflict,” which is further embedded in UNSC resolutions 1820, 1888, 1889, 1960 and 2,106. The Millennium and Sustainable Development Goals equally include goals and targets related to gender equality, which were coupled in 2010 with the creation of UN Women. These broad commitments to ensuring and mainstreaming gender equality are starting to trickle down into policymaking for animal health emergencies.

Some scholars have examined gendered issues under a One Health framework, citing gender inequities as a neglected aspect of One Health agendas (30). They cite a lack of gender-sensitive frameworks for zoonoses, human-wildlife conflict, and environmental pollution. Yet, we believe that there is evidence of change. There is a growing awareness within WOAH of the importance of gender as a determinant in both its internal and external operations. Following from WOAH’s 7th Strategic Plan, which mentions gender as an organizational ‘value’, a Gender Task Force was launched in 2021 composed of individuals from WOAH Headquarters as well as Regional/Sub-regional offices. The Gender Task Force’s objective is to contribute to WOAH’s vision on gender and also internally raise awareness of the relevance of gender to WOAH’s work. These efforts also recognize the importance of gender as a consideration in emergency preparedness and response, as reflected in the Sendai Framework for Disaster Risk Management and related international guidance documents. Moreover, the One Health Central and Eastern Africa Network, or OHCEA, has developed a training module to help advance understanding in emerging infectious disease epidemiology and outcomes with consideration for gendered roles in these outbreaks. Other researchers and practitioners have developed a framework to identify key gender considerations in One Health research with a focus on low-and middle-income countries (31), and encourage further exploration of its application related to gender-diverse populations. Moreover, there has been a self-organized Women in One Health Movement which has organically grown among women working in the sector. While these are nascent groups at present, and the latter initially focused on ensuring greater representation of women on panels and in congresses, these institutions can become powerful forces for change within the governance of animal health emergencies.

However, there was also recognition that part of the challenge is defining how else to approach the topic:

“So, I think there is like recognition that government level that something around gender needs to be done, but there's not very well articulated as to what that something ought to be”.

– National animal health agency expert, European region

## Conclusion

Thinking back to one of the first major animal health emergencies of this millennium, the 2001 FMD outbreak in United Kingdom, there has often been consideration in policy, media and informally of the major social, economic and political impacts of the epidemic, yet there is almost no consideration of gender aspects in the literature or related policy documents. The top priority of the response launched was on eliminating the pathogen ‘by massive and drastic slaughtering’, rather than considering the secondary effects such an approach may have on different sectors of society.

It may be that the historical nature of this outbreak, occurring at a time when gender was less prominent as a consideration, meant that these considerations were not even thought about. However, we can infer from the prominent literature on the economic, mental health (32) and other impacts of the outbreaks, on veterinary professionals as well as livestock producers and their families, that there likely were unrecognized gendered impacts. While it would be unfair to consider the FMD outbreak in the United Kingdom within the landscape of contemporary gendered understandings unfortunately, more recent research has continued to fail to explore gender dimensions. For example, a 2018 analysis suggested that the FMD outbreak did have a negative effect on both farmer and veterinary surgeon mental health but did not include any sex-disaggregated statistics (29). It examined suicide rates of farmers and their likelihood to leave farming as the only outcomes of these psychological effects; yet gender-based violence or other gendered impacts, which are known to be associated with human health emergencies, were not considered. The study also showed that farmers were more likely to rely on family and veterinary surgeons for support, expressing reluctance to use formal social or health services; again, based on data that most farmers in the United Kingdom are male, and assuming traditional family structures, this suggests that the emotional support burden for women may have increased. Perhaps most concerningly, there seems to have been little effort made to integrate gender considerations into modern and current policy and planning efforts related to FMD preparedness and response in the United Kingdom even if some aspects of social and economic protection have been introduced (33). This vignette highlights some of the key gendered considerations which have been missing in contemporary animal health emergency research, policy and interventions.

Understanding and mitigating the gendered effects of animal crises is not just important for drafting gender transformative policy in line with Sustainable Development Goal 5, but it can lead to a more sustainable and resilient response to animal health emergencies. Preemptively preparing for social and/or economic externalities that may emerge during a crisis can prevent and mitigate gendered harms during outbreaks. Similarly, knowing gendered social roles in animal health emergencies ensures that all labor, particularly women’s labor, is recognized, and not exploited.

Although most literature, Global North policy documents, and animal emergency experts are ignorant to the harms of gender-blind policy, there is increased recognition among leading international organizations, like WOAH, that incorporating gender into the planning process is essential to building comprehensive disaster response mechanisms. The increasing recognition that gender norms drive unique behavior and interaction with animals is a key advancement in gender awareness. Addressing harms that disproportionately impact women can be achieved through better sanitation and biosecurity education, as well as the recognition of women’s informal financial assets, like livestock in backyard farms. By providing fair compensation to women for mandatory culling of their livestock (in a form that will go directly to them, rather than a spouse, e.g., replacing the animal rather than cash payments), providing compensation for unpaid care (like subsidizing schooling or childcare), as well as supplementing biosecurity education with protective equipment when women are the primary caregiver for sick animals or people, may help reduce gendered harm.

Furthermore, recognizing the legitimacy of women’s organizations and labor union advocacy campaigns ensures that women’s labor is protected in vulnerable economic sectors by national legislation during health emergencies. Lastly, noting the importance of representation and the absence of women in decision making positions is an important consideration that must be remedied through non-discriminatory hiring practices. However, we caution against attempts to feminize the workplace which adopt an “add women and stir” mindset. The presence of women in positions of power does not directly correspond to an increase in gender transformative policy. In order to introduce better recommendations, governments must seek out gender experts–not just women–to author comprehensive gender transformative response mechanisms.

Beyond the three primary categories we have identified–representation, gendered risk, economic impact, planning must consider the mental health impacts of animal health emergencies, increased risk of gender-based violence, mental health sequelae, and as intersectionality so that primary as well as secondary features of gendered impacts in animal health emergencies can be accounted for. Our research identifies key gendered impacts of animal health emergencies that have been traditionally unexplored; however, we imagine this paper has only scratched the surface of all existing impacts. It is a starting point to seed consideration of and a push for greater research in this space.

Gender awareness is not just expanding choice or access, but fundamentally mitigating the harmful inter-relationships between resources (conditions), agency (process) and achievements (outcomes) which are inextricably linked to power (10). Together these features constitute the potential to convert choice into transformational change (10). This is of utmost importance in animal health emergencies given the high risks of economic hardship, interpersonal violence, and loss of life. Despite the aforementioned harms of gender-blind policies, there is currently insufficient research and evidence to generate meaningful guidance for decision making.

In summary, to remedy the gaps in gender-based considerations, we advance several recommendations for research and policy-making efforts: (1) collection of disaggregated data (race, gender, sexual orientation, etc.); (2) inclusion of gender experts; and (3) inclusion of primary gendered impacts (minimal representation of women in policy positions, gender roles) and secondary gendered impacts (gender-based violence, mental health, additional unpaid care responsibilities) in future planning.

Ultimately, developing policies that fairly compensate women for their time involved in emergency preparedness and response efforts will likely lead to better animal health management, as well as mitigation of the negative financial or social externalities that women and families may experience as a consequence of health emergencies. It is only by integrating gender considerations from the beginning of the planning process that we can hope to combat systemic gender blindness and build responsive, resilient systems for future animal health emergencies.

## Data availability statement

The datasets presented in this article are not readily available because primary data collected for this paper is interview data, which subject to the ethical approval is anonymous and not for broader distribution. The authors can be contacted for data access, if necessary. Requests to access the datasets should be directed to c.wenham@lse.ac.uk.

## Ethics statement

Ethical approval was obtained by the London School of Economics and Political Science Research Ethics Committee, project approval number 188842. The studies were conducted in accordance with the local legislation and institutional requirements. The participants provided their written informed consent to participate in this study.

## Author contributions

EC: Conceptualization, Data curation, Formal analysis, Funding acquisition, Investigation, Methodology, Resources, Supervision, Writing – original draft, Writing – review & editing. CS: Conceptualization, Data curation, Formal analysis, Funding acquisition, Investigation, Methodology, Resources, Supervision, Writing – original draft, Writing – review & editing. EH: Investigation, Writing – review & editing. DD: Writing – review & editing, Conceptualization, Resources. TB: Resources, Writing – review & editing. LG: Resources, Writing – review & editing. SF: Writing – review & editing. CW: Writing – review & editing, Conceptualization, Data curation, Formal analysis, Funding acquisition, Investigation, Methodology, Project administration, Resources, Supervision, Validation, Writing – original draft.

## Footnotes

^1^We note that there is a gendered difference between veterinarians, and the broader veterinary workforce which includes veterinary para-professionals, nurses and technicians.

^2^We note that gender-based violence is a Demographic and Health Survey (DHS) question.
