# Supplementary material for: Animal health emergencies: a gender-based analysis for planning and policy
Source: Front Vet Sci. 2024 Apr 5;11:1350256. doi: 10.3389/fvets.2024.1350256 (PMC11027496; doi:10.3389/fvets.2024.1350256)
Supplement: Supplementary file 1 [file Data_Sheet_1.docx]

Interview Questions (to be determined according to who being interviewed)

1. Introduction to them / their role / their work

For animal health emergency experts

1. How do you understand how policy impacts different stakeholders?
2. Have you ever thought about the gendered impacts of your work - if so how
3. Have you ever been involved in any gender mainstreaming / gender engagement process - how did it change your policy / practice / thinking etc
4. Where do you see gender in your work
   1. Representation
   2. Secondary effects of AHE policies

For Gender experts

1. How do you see the engagement with gender in the animal health emergency space?
   1. What areas of gender?
   2. What areas of AHE
2. What do you think is unique / common between how gender is considered in AHE compared to other spheres of governance?
3. What are best practice examples of gender engagement / gender mainstreaming in AHE
4. What are the enablers of gender mainstreaming in AHE
5. What are the barriers to meaningful gender engagement in AHE

Case study/disease-specific questions

ASF

- Why do you think ASF has received as much planning attention as it has?
- In your view, what have been the strengths of ASF planning?
- The weaknesses?
- It seems a lot of attention has been focused on gendered-understanding and gendered-planning with respect to ASF in Uganda. Do you think this represents something unique about ASF, or is it something culturally unique to livestock rearing in Uganda?
- What lessons can we learn from the attention paid to gender differentials and gender mainstreaming in ASF in Uganda that can be applied more generally to animal health and animal health emergencies?

FMD (2001 vs 2007)

- If UK FMD expert
  - What lessons were/were not learned between 2001 and 2007?
  - Were there considerations of gendered impacts during the 2007 response, or in after-action assessment? If not, why do you think that might have been the case?
  - Does FMD (or AHE more generally) planning in the UK now consider gender mainstreaming, and if yes, how?
    - Do you think there is scope for further gender mainstreaming?
- If not a UK person
  - What is your level of familiarity with the 2001/2007 FMD outbreaks in the UK?
  - What do you perceive to be the weaknesses that led to each becoming such large-scale emergencies?
  - Do you think there were/might have been gendered impacts to the outbreaks? If yes, what might they have been?
  - Do you have experiences of gendered impacts from other FMD outbreaks (in other countries) you have familiarity with or have been directly involved with?

HPAI

- Why do you think HPAI in SE Asia has received as much planning attention as it has?
- In your view, what have been the strengths of ASF planning?
- The weaknesses?
- It seems a lot of attention has been focused on gendered-understanding and gendered-planning with respect to HPAI in SE Asia. Do you think this represents something unique about HPAI, or is it something culturally unique to poultry rearing in SE Asia?
- What lessons can we learn from the attention paid to gender differentials and gender mainstreaming in HPAI in SE Asia that can be applied more generally to animal health and animal health emergencies?
